# Implications of vessel co-option in sorafenib-resistant hepatocellular carcinoma

**DOI:** 10.1186/s40880-016-0162-7

**Published:** 2016-11-25

**Authors:** Elizabeth A. Kuczynski, Robert S. Kerbel

**Affiliations:** Sunnybrook Research Institute, 2075 Bayview Avenue, S-Wing, Room S217, Toronto, ON M4N 3M5 Canada

**Keywords:** Vessel co-option, Non-angiogenic, Resistance, Sorafenib, Hepatocellular carcinoma

## Abstract

The reason why tumors generally have a modest or transient response to antiangiogenic therapy is not well understood. This poses a major challenge for sorafenib treatment of advanced hepatocellular carcinoma (HCC) where alternate therapies are lacking. We recently published a paper entitled “Co-option of liver vessels and not sprouting angiogenesis drives acquired sorafenib resistance in hepatocellular carcinoma” in the *Journal of the National Cancer Institute*, providing a potential explanation for this limited benefit. We found that in mice bearing HCCs that had acquired resistance to sorafenib, tumors had switched from using angiogenesis for growth to co-opting the liver vasculature by becoming more invasive. Accumulating evidence suggests that many human tumor types may use vessel co-option, which has profound implications for the use of anti-angiogenic agents for cancer treatment.

## Background

Advanced hepatocellular carcinoma (HCC) is difficult to treat. It is a highly aggressive tumor, intrinsically resistant to conventional anti-cancer therapies such as chemotherapy and irradiation. Treatment options were bleak until 8 years ago when the oral kinase inhibitor sorafenib (Nexavar) was approved for advanced HCC treatment, yet sorafenib remains the only approved systemic drug treatment.

Sorafenib is a selective inhibitor of vascular endothelial growth factor receptor 2 (VEGFR2), platelet-derived growth factor receptor (PDGFR), and Raf kinases, and its anti-tumor mechanism includes stromal effects such as anti-angiogenesis and direct anti-proliferative and pro-apoptotic effects on tumor cells [1]. HCCs are hypervascular tumors. However, the durability of response to sorafenib is limited, with median extensions in overall survival of 2–3 months and single-digit response rates [2, 3]. Other more potent or specific VEGF-targeted agents have been proved to be no better than sorafenib in randomized phase II or III clinical trials or have failed to improve outcomes as second-line therapy [4]. Sorafenib resistance therefore remains a major clinical challenge, which cannot be controlled by anti-VEGF pathway angiogenesis inhibitors alone.

## Vessel co-option as a resistance mechanism

**Fig. 1:**
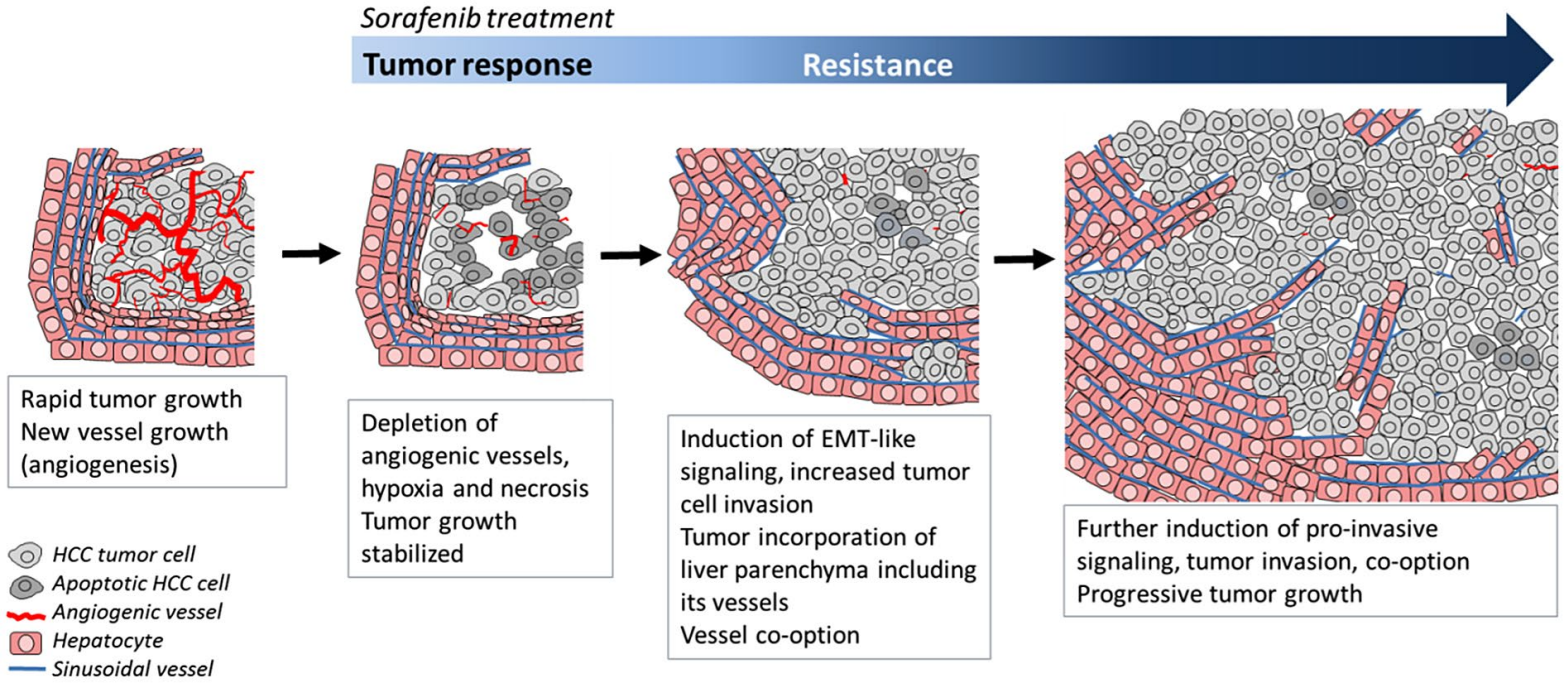
Proposed mechanism of acquired resistance to the multikinase inhibitor sorafenib in hepatocellular carcinoma (HCC). Highly angiogenic HCCs are initially responsive to sorafenib treatment. Over time, tumor cells become more invasive which promotes co-option of liver vessels in the face of angiogenesis blockade. *EMT* epithelial-to-mesenchymal transition

In our study “co-option of liver vessels and not sprouting angiogenesis drives acquired sorafenib resistance in hepatocellular carcinoma” recently published in the *Journal of the National Cancer Institute* [5], we proposed a new explanation for why the anti-tumor effects of sorafenib on HCC may be short-lived and why alternate anti-VEGF agents, especially as second-line therapies, are not helping (Fig. 1). We found that orthotopically grown HCCs in immune-compromised mice were highly angiogenic and that daily sorafenib treatment initially potently depleted angiogenic vessels and significantly slowed tumor progression. However, tumors began to manifest signs of drug resistance after being treated for a month. At this time, tumor cells began to grow in a highly invasive manner but did not induce new blood vessel growth. Rather, the tumor cells had surrounded the sinusoidal and major vessels of the liver and incorporated them into its mass. Since these liver vessels that were hijacked by the tumor (“co-opted” from the host) were originally “normal” vessels, they could not be blocked by sorafenib treatment. Only when sorafenib treatment was discontinued, tumors showed fewer signs of invasion and switched back to relying on angiogenesis.

The finding that certain tumors can grow along and co-opt the normal tissue vasculature without inducing neo-angiogenesis has been under-appreciated, if not ignored. Vessel co-option has been found to be a mode of vascularization in several human tumor types which grow in vessel-rich organs, including the brain, lungs, and liver [6]. These organs offer an appealing environment for tumor growth due to a high oxygen and nutrient supply. Certain tumors innately “prefer” using angiogenesis over co-option, or the converse, and others use both vascular mechanisms simultaneously. Vessel co-option may be important at early stages of HCC progression prior to induction of capillarization (angiogenesis) [7] and in well-differentiated HCCs in which vessels have been found to express markers of sinusoidal liver endothelial cells [8]. In the absence of anti-angiogenic treatment, advanced HCCs were found to have growth patterns consistent with vascularization through either angiogenesis by growing as encapsulated masses, or vessel co-option by infiltrating the sinusoids or replacing the hepatic cords [9]. There is therefore a strong possibility that depleting angiogenic vessels could cause an HCC to switch to dependence on the co-opted liver vessels. Potentially, some HCC patients may be resistant to sorafenib upfront because their tumors use mostly co-option, or, as we observed in mice, their tumors switch to this mechanism.

## Treatment-induced invasion and EMT

The role of vessel co-option during sorafenib resistance may be connected to the finding that, in certain situations, treatment of mice with anti-angiogenic agents can render cancer cells more invasive and metastatic [10, 11]. This may occur secondary to increased hypoxia in tumors caused by the anti-angiogenic effects of the drug. For example, increased rates of metastasis and local tumor cell invasion were observed in pancreatic neuroendocrine tumor- and glioblastoma-bearing mice after treatment with anti-VEGFR2 monoclonal antibody or sunitinib [11]. This occurred despite primary tumor shrinkage and depletion of angiogenic vessels, similar to what we observed in HCCs during sorafenib treatment. One could speculate that during anti-angiogenic treatment, the invading fronts of such tumors contained instead the co-opted local vessels of the pancreas and brain, but this is currently unknown.

Further underscoring the mechanism that increased HCC invasion precipitated vessel co-option, we used microRNA screening and reverse transcription-polymerase chain reaction to find that the epithelial-to-mesenchymal transition (EMT) pathway corresponded to the onset of invasion in HCC tumors [5]. EMT is considered an important pathway for the early steps of tumor invasion and metastasis, during which tumor cells lose their polarity, adopt a mesenchymal morphology, and become more motile [12]. EMT has previously been proposed as a mechanism of sorafenib resistance in HCC cells in vitro by gradually increasing the concentration of sorafenib over months [13, 14]. Thus, resistant HCC cells appeared mesenchymal, up-regulated several EMT genes, were more invasive and more metastatic once implanted into mice relative to parental cell lines [13, 14]. By in vivo treatment, we observed significant changes in vimentin, Zeb1/2, and E-cadherin expression, but HCC cells remained epithelial in morphology and were not more metastatic. The apparent differences in the behavior of HCC cells between in vivo and in vitro treatments of HCC, the latter involving more prolonged treatment and supra-physiologic drug concentrations, are notable when considering the translation of these findings to the clinic.

The pro-invasive/metastatic effects observed in mice have caused some debates because accelerated disease progression (such as an increased rate of metastasis) has not been observed in large follow-up studies of patients treated with sunitinib or bevacizumab [15, 16]. These conflicting clinical/preclinical results could be explained if a major consequence of anti-angiogenic treatment-induced invasion is not metastasis, but rather an increased tumor reliance on vessel co-option. The above clinical studies unfortunately cannot inform on potential pro-invasive effects that occur *on*-*treatment* because data was collected long after anti-angiogenic therapy discontinuation [17] and potential reversal of vascular changes.

## Future research

Specific molecular markers of co-opted vessels are presently unknown. Therefore, histological analyses of tumor growth patterns as well as expression of angiogenic and tissue-specific endothelial and epithelial cell markers are necessary for insight into the occurrence of vessel co-option in tumors. Such analyses of the vascular phenotype during long-term sorafenib treatment of HCCs are highly deficient. Rather, in vitro studies of HCC cell lines predominate the literature [18] which disregards the stromal effects of sorafenib.

Our finding needs to be confirmed in HCC patients. The procurement of patient tumor specimens of sufficient volume while a patient is on anti-angiogenic therapy is a major challenge but is highly needed. We are aware of a single report in which biopsies of an HCC patient’s tumor obtained prior to and during disease progression on sorafenib were screened for resistance mechanisms. Proteomic and phospho-proteomic analysis strongly implicated EMT and anti-adhesive signaling during sorafenib treatment or resistance [19]. Further hints that vessel co-option mediates clinical resistance to anti-angiogenic therapy was published very recently in a retrospective study, in which a “replacement” growth pattern involved co-option of vessels with poor clinical responses to bevacizumab in liver metastases [20].

Vessel co-option is increasingly realized as a major blood supply for tumors which have crucial implications for therapy selection. If vessel co-option is proved to be a cause of clinical drug resistance in HCC, the use of “anti-vascular” rather than anti-angiogenic therapies will be an important therapeutic strategy going forward. Drugs which block or inhibit tumor cell invasion when this process is known to precede a switch to vessel co-option by tumors, may be evaluated as an alternative therapy.
